# Comparison of Multiple Displacement Amplification (MDA) and Multiple Annealing and Looping-Based Amplification Cycles (MALBAC) in Limited DNA Sequencing Based on Tube and Droplet

**DOI:** 10.3390/mi11070645

**Published:** 2020-06-29

**Authors:** Xiaoxiang Zhou, Ying Xu, Libo Zhu, Zhen Su, Xiaoming Han, Zhen Zhang, Yan Huang, Quanjun Liu

**Affiliations:** State Key Laboratory of Bioelectronics, School of Biological Science and Medical Engineering, Southeast University, No. 2, Sipailou, Nanjing 210096, China; 230189166@seu.edu.cn (X.Z.); 220174594@seu.edu.cn (Y.X.); 230169449@seu.edu.cn (L.Z.); 220174564@seu.edu.cn (Z.S.); 220171812@seu.edu.cn (X.H.); 220181842@seu.edu.cn (Z.Z.); 101010225@seu.edu.cn (Y.H.)

**Keywords:** whole genome amplification, droplets, multiple displacement amplification, multiple annealing and looping based amplification cycles

## Abstract

Whole genome amplification (WGA) is crucial for whole genome sequencing to investigate complex genomic alteration at the single-cell or even single-molecule level. Multiple displacement amplification (MDA) and multiple annealing and looping based amplification cycles (MALBAC) are two most widely applied WGA methods, which have different advantages and disadvantages, dependent on research objectives. Herein, we compared the MDA and MALBAC to provide more information on their performance in droplets and tubes. We observed that the droplet method could dramatically reduce the amplification bias and retain the high accuracy of replication than the conventional tube method. Furthermore, the droplet method exhibited higher efficiency and sensitivity for both homozygous and heterozygous single nucleotide variants (SNVs) at the low sequencing depth. In addition, we also found that MALBAC offered a greater uniformity and reproducibility and MDA showed a better efficiency of genomic coverage and SNV detection. Our results provided insights that will allow future decision making.

## 1. Introduction

High throughput sequencing has tremendously influenced biomedical research due to its ability to acquire massive amounts of sequence data [1,2,3]. Whole genome sequencing has investigated the characteristic of tumor evolution [4,5], microbial infection [6,7], and neurodevelopment [8]. Whole genome sequencing usually requires DNA samples at nanogram to microgram levels [9,10]. However, uncultured microbes isolated from environmental samples generally only contain femtogram or picogram DNA [10,11,12]. The analysis of clinical samples frequently include only a limited amount of DNA [13,14]. Thus, it is necessary to amplify DNA to adequate quantity without altering the representation of the original DNA. WGA included two categories: based on the polymerase chain reaction (PCR) and MDA method [15,16]. In general, MDA and MALBAC are two most widely applied WGA methods, which have different advantages and disadvantages dependent on the research objectives. MDA generates a sufficient quantity of replicated DNA with high fidelity and large fragment size (10–20 kb), however, it is troubled with amplification bias which results in a different coverage [10,17,18], and DNA contaminating is also a major problem. MALBAC uses random priming and poikilothermic preamplification in the early stage, followed by PCR amplification with limited circles [19,20]. It suppresses the random bias of amplification and exhibits reduced allelic dropout rate [20,21], coverage and uniformity are both improved by virtue of quasilinear amplification intermediately [22]. These approaches can be performed combining with the microfluidic method, which has extensively been applied to genomics [23,24], proteomics [25,26], metabonomics [27,28]. Microfluidics provide powerful and flexible platforms for MDA and MALBAC, integrating labor-intensive experimental processes in an individually closed device [18,21,29,30]. In particular, droplet microfluidics provides a closed chemical reaction environment by emulsification [31], and has been used to identify complex single-cell phenotypes [23], to detect nucleic acid of pathogenic bacteria [32], and to profile the genomics at high throughput [33]. Recently, some groups combined droplet microfluidics with MDA or the MALBAC technique for bias-less single-cell WGA [10,17,18,21,31,34], which can get higher quality of WGAs than conventional methods. The droplet method can possibly be better when tiny amounts of limited genome can be amplified.

In this paper, we performed the comparison of tube MDA (tMDA), tube MALBAC (tMALBAC), droplet MDA (dMDA), and droplet MALBAC (dMALBAC) using limited DNA samples to give more insights of these different approaches, which will be helping the future decision making regarding WGA protocols. We preliminarily estimated the efficiency of amplification using agarose gel electrophoresis and fluorescence. Through further sequencing analysis, we demonstrated that droplets are able to effectively decrease the non-uniformity and amplification bias than tubes. Furthermore, we found that MALBAC significantly improved the uniformity and reproducibility while MDA shown a better efficiency in genomic coverage and SNV detection. Our results can provide a guidance to choose a better method.

## 2. Materials and Methods

### 2.1. Device Fabrication

The droplets generated microfluidic device was designed by AutoCAD software and fabricated using conventional UV lithography techniques [35]. The width and height of the microchannel was 100 and 40 μm, respectively, and the width and length of the intersection were 50 μm. The photomask pattern (Mask customization company, Shenzhen China) was transferred to a layer of negative photoresist (SU8-2025, Microchem, Newton, MA, USA) and then coated on a silicon wafer (Prime, GuijingTec, Shenzhen, China) at 2000 rpm for 30 s using a spinner (TT, SÜSS MICROTEC SE, Garching, Germany). The processed wafer was baked in a vacuum oven (DZF-6032, BluePard Corp, Shanghai, China) at 65 °C for 3 min and then at 95 °C for 5 min. The photolithography mask (Mask customization company, Shenzhen, China) and the wafer were put into the mask aligner (MA6, SÜSS MICROTEC SE, Garching, Germany) and then exposed to UV light for 12 s. The wafer was baked again and then dipped in SU-8 developer solution (MICRO CHEM, Newton, MA, USA) for 5 s. After developing the microstructure, a 10:1 (w/w) mixture of poly (dimethylsiloxane) (PDMS) with curing agent (Sylgard 184 silicone elastomer kit, Dow corning, Midland, MI, USA) was poured on the pattern and cured for 2 h at 80 °C, bubbles in mixture were removed by vacuum. After the solidified PDMS molds were peeled, the channel inlets and outlets were punched with a 0.75 mm biopsy punch (WENHAO, Corp, Suzhou, China) for connection to pumps via PTFE tubing (BEION Corp, Shanghai, China). Then, the PDMS replica and glass slide were treated by plasma machine (PDC-002, HARRIC SCIENTIFIC Corp, New York, USA) and then bonded together at 80 °C for 1 hour. Finally, the microchannel was filled with Aquapel solution (PPG Industries, Pittsburgh, PA, USA) to produce a hydrophobic surface coating and then baked at 65 °C for 5 min.

### 2.2. Whole Genome Amplification

YH-1 cells, the immortalized cells of a Chinese individual, were provided by BGI Shenzhen (Shenzhen, China). Genomic DNA of YH-1 cells was extracted using a QIAamp DNA Mini Kit (Qiagen) and then quantified by the Qubit 2.0 system (Thermo Fisher Scientific, Waltham, MA, USA). In this paper, we compared the conventional tube WGA with droplet WGA using two commercialized WGA kits. In the tube method, a REPLI-g Midi Kit (Qiagen) of MDA was applied to amplify 12.500 ng samples in a 50 μL system consisting of 7 μL nuclease-free water, 31 μL reaction buffer, 2 μL DNA polymerase, and 10 μL templates. After 3 h of incubation at 30 °C, the enzyme was inactivated at 65 °C for 5 min. A MALBAC® Single Cell DNA Quick-Amp Kit (Yikon Genomics, Shanghai, China) of MALBAC was applied to amplify 12.500 ng samples in a total volume of a 72 μL system consistent of 15 μL nuclease-free water, 45 μL Rap-WGA solution, 2 μL RWGA Enzyme Mix, and 10 μL templates, while reaction condition was 95 °C for 3 min, then ran 10 cycles of preamplification (for each cycle: 20 s at 10 °C, 30 s at 30 °C, 40 s at 50 °C, 2 min at 70 °C, 20 s at 95 °C, and 10 s at 58 °C), followed with 95 °C for 3 min and ran 21 cycles of PCR amplification (for each cycle: 20 s at 94 °C, 15 s at 58 °C, and 2 min at 72 °C). Furthermore, in the droplet method, 20× Evagreen (Biotium, Fremont, CA, USA) was particularly added into the MDA and MALBAC system for monitoring of WGA products, other parts of the droplet system and reaction condition were the same with the tube method. Mixed mineral oil (Sigma Aldrich) with 3% (w/w) surfactant (ABIL-EM90, EVONIK, Essen, Germany) and 0.2% (w/w) Triton X-100 (Sigma Aldrich, Louis, MO, USA) as continuous phase to generate droplets. Droplets were generated from the intersection, at flow rates of 30 μL/h for dispersed-phase liquids and 150 μL/h for the carrier oil. All reaction systems were mixed slightly but completely by vortex and then loaded into the microfluidic device. 

### 2.3. Quality Assay Based on Fluorescence and Electrophoresis

We preliminarily estimated the efficiency of amplification using agarose gel electrophoresis and fluorescence. We designed the initial templates with 12.500 ng to investigate the efficiency of WGA in droplets based on fluorescent readout, without a template as a negative control. Collected droplets were transferred on the glass slide for microscopic observation. Bright-field and fluorescent images were observed and then captured using a fluorescence microscope (Axio Vert A1, ZEEISS, Oberkochen, Germany) integrated with a digital camera. The diameter and fluorescence intensity of droplets were calculated by using ImageJ software. We synchronously achieved WGA in tubes using the same templates. 

After observation, the droplets firstly were demulsificated by isopropanol (80109218, SCR Corp, Shanghai, China), and then recycled by DNA Clean & Concentrator Kit (ZYMO RESEARCH, Irvine, CA, USA), followed, quantified by using a Qubit 2.0 system. Afterwards, 1% agarose gel electrophoresis was carried out to separate and analyze the WGA products produced by the droplet and tube methods. The remaining products were stored at −20 °C. 

### 2.4. Library Preparation and Whole Genome Sequencing

After quantification, the tMDA, tMALBAC, dMDA, and dMALBAC libraries were prepared by using the MGIEasy DNA Library Rapid Prep Kit (MGI, Shenzhen, China), while the initial templates were 12.500 ng. The resulting libraries were size-checked by using an Agilent 2100 Bioanalyzer system (Agilent). All libraries were sequenced by the MGISEQ-2000 platform with 150 bp pair-end reads. Sequencing data with eligible quality were mapped to the hg19 reference genome using the BWA-MEM alignment algorithm [36].

### 2.5. Sequencing Analysis

Before the analysis of sequencing results, the acquired reads were normalized to 1×, 2×, 4×, 6×, 8×, 10×, 13×, 16×, 20×, 25×, 30×, and 40× coverage per each genome for each sample. The quality and reliability of the original data was evaluated by using FastQC [37]. Mapped reads were sorted and piled up using SAMtools [38] and statistically analyzed with QualiMap2 [39]. After removal of duplications and secondary reads, the uniquely mapped reads were respectively assigned to bins of fixed size for coverage and uniformity analysis. In addition, reads were imported into Ginkgo software [40] which used variable-sized bins for copy number variations (CNVs) identification.

## 3. Results and Discussion

### 3.1. Overview of the Method

**Figure 1:**
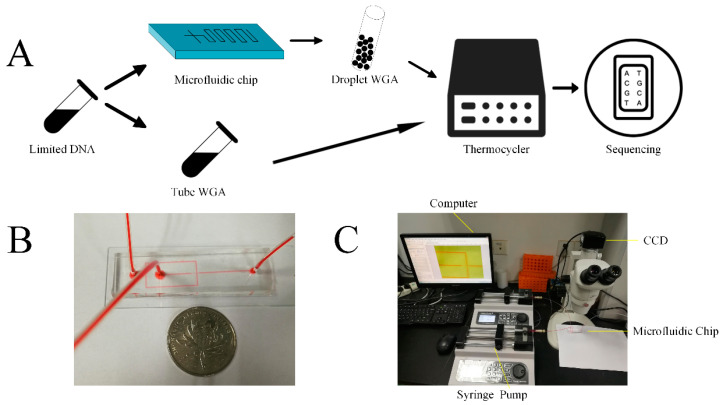
Schematic illustration of experimental process and setup. (**A**) MDA and MALBAC ready YH-1 genomic samples were partitioned into picoliter droplets using a microfluidic device. The generated MDA droplets and tMDA were incubated at 30 °C for 12 h. The generated MALBAC droplets and tMALBAC were amplified by following the manufacture’s protocols. DNA amplicons were then purified, cleaned, and prepared for the following sequencing. (**B**) Photograph of a microfluidic channel filled with red ink. (**C**) Schematic illustration of experimental setup for the droplets generation and observation.

In order to compare the performance between MDA and MALBAC comprehensively and quantitatively, we performed MDA and MALBAC in droplets and tubes within limited samples synchronously, and then sequenced and analyzed the products. This workflow was illustrated in Figure 1A. We fabricated a PDMS microfluidic device for generating picoliter droplets (Figure 1B). In order to address both the bias in amplification and non-specific amplification, we emulsified the reaction mixture containing 12.500 ng DNA samples. After incubation, the products were accumulated in individual droplets, resulting in the fluorescent products throughout in the droplets. The process of droplets generation was achieved in our microfluidic system (Figure 1C). The amplified whole genomes were collected and sequenced to compare MDA and MALBAC deeply. To quantify the effectiveness, amplification uniformity and SNVs of the four sequencing results were profiled. We found that whole genome sequencing in the droplets amplification are better than the traditional tube method. Overall, MALBAC significantly improved the uniformity and reproducibility while MDA showed a better efficiency in genomic coverage and SNV detection. 

### 3.2. Whole Genome Amplification in Droplets

**Figure 2:**
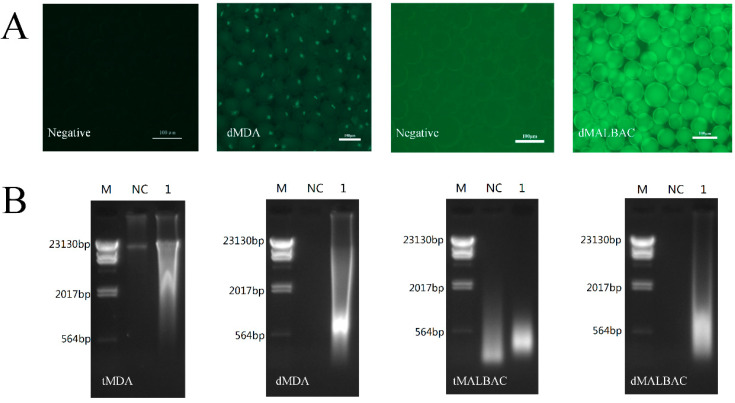
WGA of limited YH-1 genomic samples. (**A**) Fluorescent images of dMDA and dMALBAC encapsulating 12.500 ng initial samples. The droplets without samples were negative control. (**B**) 1% Agarose gel electrophoresis of WGA products. M, DNA molecular weight markers; NC, negative control; Lane 1, amplified DNA samples.

**Figure A1:**
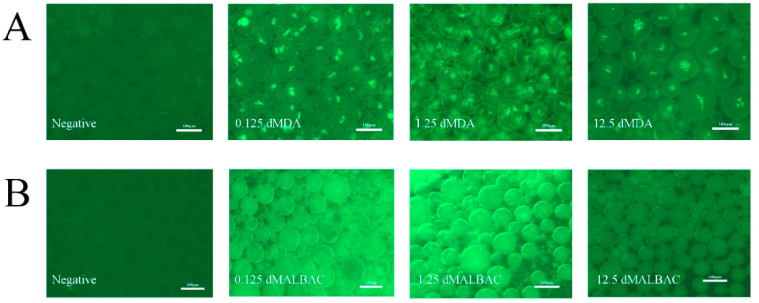
The quality-dependent changes in the fluorescence intensities of DNA-intercalating dye in dMDA and dMALBAC. (**A**) Fluorescent images of dMDA encapsulating 0.125 ng, 1.25 ng and 12.5 ng initial samples with Evagreen dye, respectively. The droplets without samples were negative control. (**B**) Fluorescent images of dMALBAC encapsulating 0.125 ng, 1.25 ng and 12.5 ng initial samples with Evagreen dye, respectively. The droplets without samples were negative control.

To validate that the droplet system enabled massively parallel to generate reaction environments within limited DNA samples, MDA and MALBAC mixtures were emulsified with 12.500 ng DNA and without DNA, respectively. After incubation, the appearance of fluorescence was successfully observed in droplets which reacted within DNA samples (Figure 2A). In the meantime, the dimensions and fluorescent intensities were uniform among individual droplets, which suggested that microfluidic droplets enabled to amplify genome within individual droplets from a single DNA molecule [17]. We demulsified and collected the amplified whole genomes, and performed agarose gel electrophoresis. The results illustrated that the range of the WGA products’ sizes were approximately 0.3–2.0 kb in MALBAC and 0.5–20.0 kb in MDA, respectively (Figure 2B). The analysis demonstrated that the amplification in droplets can effectively eliminate contaminations which frequently appeared in the traditional tube reaction. To further demonstrate the ability of WGA in droplets, the reaction mixture emulsified with 0.125 and 1.250 ng templates were also carried out, and the quality-dependent changes in the fluorescence intensities of amplified DNA in each droplet were shown in Figure A1. The dMDA with 12.500 ng templates showed similar fluorescence intensities with 1.250 ng templates, while 0.125 ng templates appeared an obviously weaker fluorescence. However, the dMALBAC with 3 types of initial templates showed similarly strong fluorescence intensities, which performed a better efficiency of amplification.

### 3.3. Coverage Breadth and Genome Recovery

**Figure 3:**
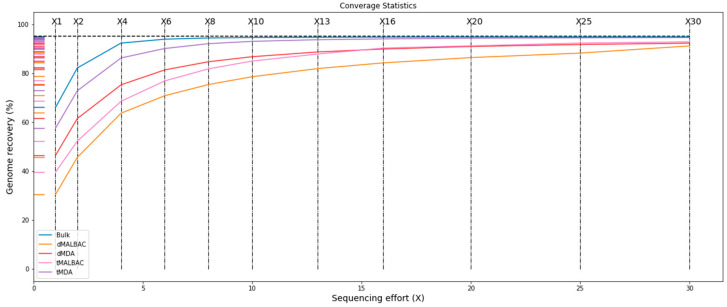
Deep-sequenced data analysis showing recovery of tMDA, dMDA, tMALBAC, and dMALBAC methods.

We found that the parallel assays revealed similar performance. Then, we sequenced tMDA, tMALBAC and parallel dMDA, dMALBAC assays respectively by using 12.500 ng genomic DNA as input, and unamplified 1 µg bulk YH-1 genomic DNA was a reference. According to the 30× deep-sequenced data, we found that genomic average depth using the droplet method was higher than traditional tube amplification, and MDA was obviously higher than MALBAC (Table 1). It indicated that the higher genomic average depth resulted from the effect of bias suppression in compartmentalized reactions. The read mapping ratio and genome coverage was similar in the four products. Additionally, the analysis of different deep-sequenced data generally showed that genomic recovery of MDA products was higher than MALBAC, and the genomic recovery of the conventional tube method was higher than the droplet method (Figure 3). Moreover, this phenomenon was more evident when comparing the coverage of genome in low sequencing depth. When the sequencing depth was more than 15×, the genomic recovery of the four products increased stably. 

### 3.4. Amplification Uniformity and SNV Detection

**Figure 4:**
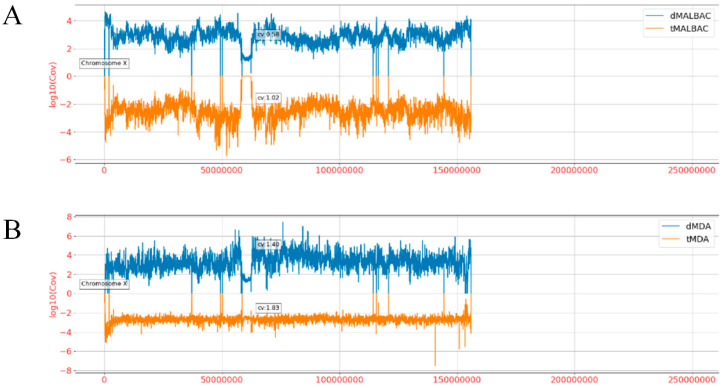
Comparison of sequencing coverage between tube and droplet reads over the entire chromosome X. (**A**) The standardized mean coverage depth of dMALBAC reads (blue bars, above X axis) and tMALBAC reads (orange bars, below X axis) were calculated. (**B**) The standardized mean coverage depth of dMDA reads (blue bars, above X axis) and tMDA reads (yellow bars, below X axis) were calculated.

**Figure A2:**
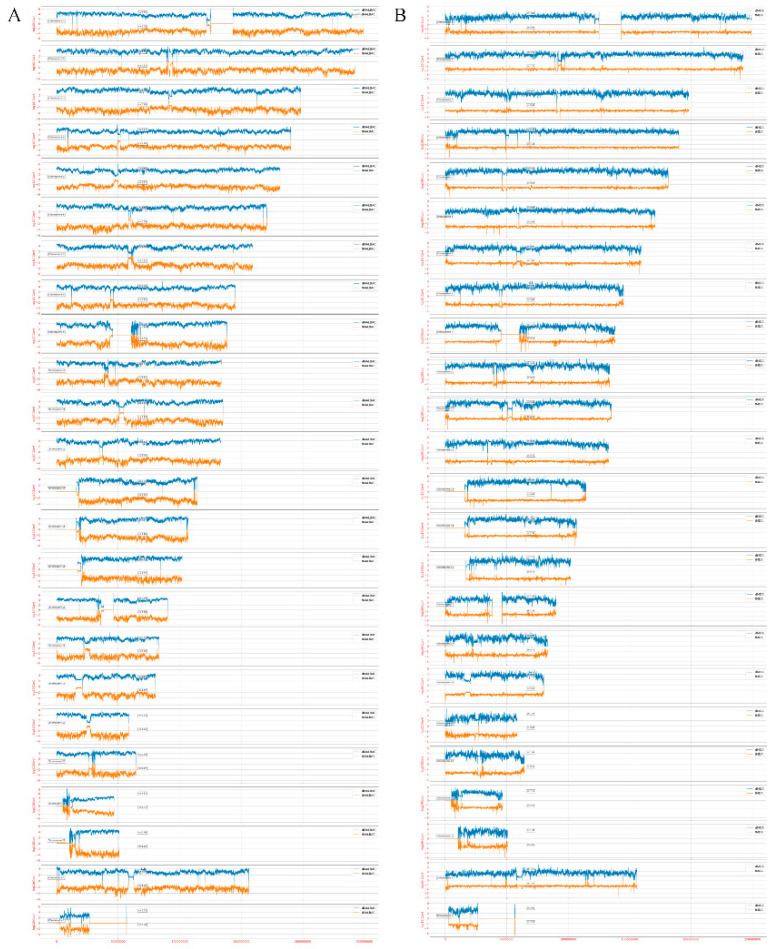
Comparison of sequencing coverage between tube reads and droplet reads. (**A**). The standardized mean coverage depth of dMALBAC reads (blue, above X axis) and tMALBAC reads (orange, below X axis) in each Chromosome were calculated. (**B**) The standardized mean coverage depth of dMDA reads (blue, above X axis) and tMDA reads (orange, below X axis) in each Chromosome were calculated.

**Figure A3:**
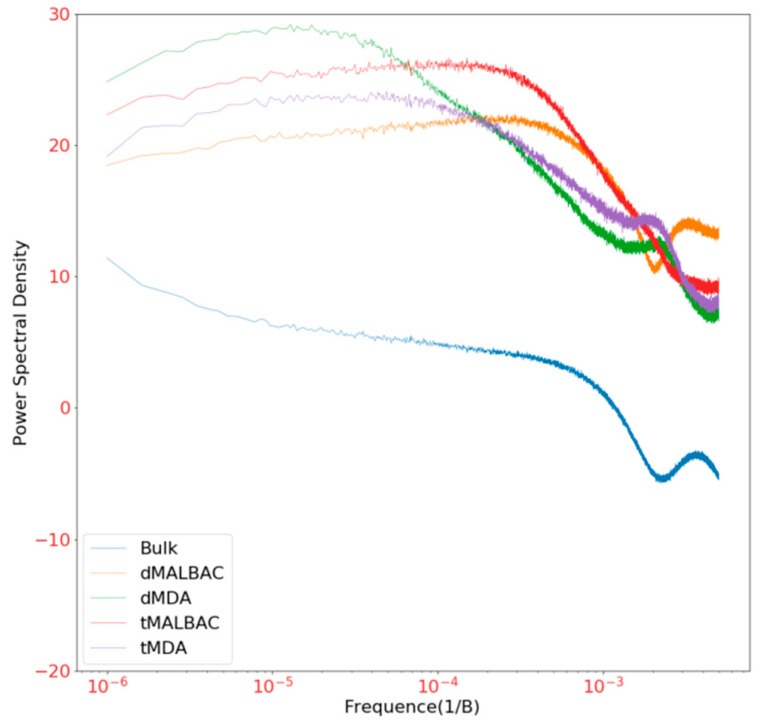
The power spectra of read density based on the sequencing result using tMDA, dMDA, tMALBAC and dMALBAC methods were plotted to validate the coverage uniformity of genome.

To investigate the genomic coverage distribution, the whole genome of hg19 was separated into bins of fixed 40 kb size, and then the standardized average depth of each bin was calculated [29]. To exclude the specificity of four samples, read depth of each bin was normalized by unamplified bulk data. We found that the coverage distribution of dMDA reads was significantly higher than tMDA reads in the whole genome, and dMALBAC reads higher than tMALBAC, respectively (Figure A2). From the histogram of the read depth over the entire chromosome X (Figure 4A), it was shown that the CV of dMALBAC sequencing data was generally 0.58, while the tMALBAC sequencing data with CV was 1.02. The CV of dMDA sequencing data was generally 1.40, while the tMDA sequencing data with CV was 1.83 (Figure 4B). The superiority of uniformity in the droplet method was significantly prominent than the traditional tube method. The power spectra of read density based on the sequencing result were also plotted to validate the coverage uniformity of the genome (Figure A3). The analysis confirmed that droplet method showed better uniformity than the tube method due to the effectively suppressed amplification bias.

In terms of SNV detection, MDA showed a better detection rate than MALBAC on both homozygous and heterozygous SNVs (Table 2). The highest detection rate with SNVs was 74.97% by tMDA. However, the allelic dropout (ADO) rate of MALBAC was lower than MDA, the tube method was lower than the droplet method. Furthermore, the error rate of MDA was superior to MALBAC. It agreed with previous reports [41]. Moreover, the false positive rate of dMALBAC (19.96%) was the lowest among the four methods. When the input data was reduced to 10×, it showed that the droplet method achieved an obviously higher detection rate, which means that the droplet method was more sensitive than the conventional tube method in a low sequencing depth (Table 3). 

## 4. Conclusions

Whole genome sequencing is an increasingly popular approach in biology and medical research which has been applied widely in analysis of genetic and metabolic diversity with environmental and clinical samples [3,42,43]. The whole genome amplification strategy is imperative for whole genome sequencing which necessitates significant amplification of the target genome in preparation of sequencing libraries [44,45]. It is necessary that tiny amounts of limited genome can be amplified without contamination in a high-throughput manner. MDA has widely been applied to detect nucleic acid [46,47,48], and MALBAC currently has been applied to analyze genomics. The droplet method applies emulsion to divide the DNA fragments into a large number of aqueous droplets and drives the amplification to saturation in each droplet [17,34]. There are already lots of applications of MDA based on droplets [17,18,44]. However, the applications of MALBAC combined with droplets has not been reported.

In this paper, we found that MDA and MALBAC in droplets was highly effective even though the initial templet was 125 pg (~20 cells) based on the fluorescent readouts. Furthermore, dMDA and dMALBAC could dramatically reduce the amplification bias and retain the high accuracy of replication than tMDA and tMALBAC. In terms of SNV detection, the droplet method showed higher efficiency and sensitivity for both homozygous and heterozygous SNVs in low sequencing depth. Therefore, which indicated the droplet method has the advantage in extending the understanding of genomic diversity. In addition, MALBAC showed higher uniformity and reproducibility than MDA, which was consistent with previous reports [49,50], and droplets can further improve the efficiency of uniformity and reproducibility. However, MDA showed a better efficiency of genomic coverage and SNV detection. 

MALBAC and MDA methods have different advantages and disadvantages, which can be selected according to different research objectives. Generally, the amplification conditions such as reaction time and reagent components are crucial, which should be optimized carefully to reduce the bias and obtain more genome coverage. Our results provided insights that will allow future decision making regarding WGA protocols. 

## Figures and Tables

**Table 1:** Deep-sequencing statistics of limited DNA amplified by tMDA, dMDA, tMALBAC, and dMALBAC methods.

Sample/Method	Read Mapping Ratio (%)	GC Content (%)	Mean Depth (×)	Genome Coverage (%)
bulk	99.67	40.87	30.91	94.83
tMDA	99.65	41.41	30.84	94.55
dMDA	99.69	38.76	43.62	92.94
tMALBAC	99.61	47.13	29.96	92.82
dMALBAC	99.45	46.13	40.11	91.11

**Table 2:** Summary of the comparison between different methods for SNV detection on chromosome 1 of normal diploid YH-1 cell based on sequencing data of larger than 30× data size.

Parameter	Sample Type				
	dMDA	tMDA	dMALBAC	tMALBAC	Bulk
Total SNVs	24,2393	32,5728	22,6516	21,9911	43,4452
Detection rate	55.79%	74.97%	52.14%	50.62%	N/A
Heterozygous SNVs	124,015	153,704	111,856	122,428	165,177
Detection rate	75.08%	93.05%	67.72%	74.12%	N/A
Homozygous SNVs	118,378	172,024	114,660	97,483	269,275
Detection rate	43.96%	63.88%	42.58%	36.20%	N/A
ADO rate	1.58%	1.22%	0.92%	0.52%	N/A
SNV error rate	0.002%	0.002%	0.06%	0.049%	N/A
False-positive rate	25.13%	25.03%	19.96%	26.05%	N/A

**Table 3:** Comparison of SNV-detection efficiency between tube and droplet methods using 10× sequencing data.

Parameter	Heterozygous SNVs	Homozygous SNVs	Total SNVs
Bulk (30×)			
SNVs	165,177	269,275	434,452
Bulk (10×)			
SNVs	153,212	165,070	318,282
Detection rate	92.76%	61.30%	73.26%
dMDA (10×)			
SNVs	52,325	15,005	67,330
Detection rate	91.135%	69.525%	80.330%
tMDA (10×)			
SNVs	103,535	101,481	205,016
Detection rate	62.68%	37.69%	47.19%
dMALBAC (10×)			
SNVs	69,417	66,793	136,210
Detection rate	42.02%	24.80%	31.35%
tMALBAC (10×)			
SNVs	67,410	47,705	115,115
Detection rate	40.81%	17.72%	26.50%
